# Optimization of the Cold Water Extraction Method for High-Value Bioactive Compounds from Chamomile (*Matricaria chamomilla* L.) Flower Heads Through Chemometrics

**DOI:** 10.3390/molecules29204925

**Published:** 2024-10-17

**Authors:** Martina Foschi, Lorenzo Marsili, Ilaria Luciani, Giulia Gornati, Claudia Scappaticci, Fabrizio Ruggieri, Angelo Antonio D’Archivio, Alessandra Biancolillo

**Affiliations:** Department of Physical and Chemical Sciences, University of L’Aquila, Via Vetoio, Coppito, 67100 L’Aquila, Italy

**Keywords:** chamomile, design of experiment (DOE), 2,2-diphenyl-1-picrylhydrazyl (DPPH), high-performance liquid chromatography–diode array detector (HPLC-DAD), ultraviolet–visible spectroscopy (UV-Vis), cold water extraction

## Abstract

This study focused on optimizing a cold water extraction method to obtain bioactive compounds from chamomile (*Matricaria chamomilla* L.), addressing increasing consumer demand for natural products and nutraceuticals. A full-factorial design was employed to evaluate the effects of temperature, time, and chamomile amount on the polyphenolic profile of extracts. The samples were characterized by HPLC-DAD and UV-Vis coupled with chemometrics; the analysis showed that extraction time negatively affected extract quality, as did the interaction between time and temperature. In addition, a significant positive quadratic effect for temperature and a positive coefficient for chamomile amount was found. ASCA was used to assess the UV-Vis profile, offering an alternative untargeted method for understanding the variable effects. The optimal extraction conditions (25 °C, 32 min, and 2.5 g of chamomile) produced samples high in hydroxybenzoic and hydroxycinnamic acids and flavanol derivatives. Using A face-centered design, this study also monitored antioxidant activity via a DPPH scavenging assay, confirming that the optimal conditions yielded samples within the range of maximum antioxidant activity in the studied experimental domain.

## 1. Introduction

Chamomile, particularly *Matricaria chamomilla* L., is a widely used herb with significant therapeutic properties, including anti-inflammatory, analgesic, and sedative effects [1]. Belonging to the Asteraceae family, chamomile is an important component in phytotherapy [2] and is thus included in numerous pharmacopeias and pharmaceutical, cosmetic, and food applications [3,4]. Increasing industrial demand has spurred research into the safety and bioactivity of chamomile formulations [5,6,7,8].

In this context, there is growing interest in exploring unconventional chamomile extraction methods that are environmentally friendly and minimize sample degradation. Supercritical fluid extraction (SFE) using carbon dioxide excels in extracting lipophilic fractions rich in essential oils, albeit requiring complex and costly equipment [9]. Microwave-assisted and ultrasonic extractions are also considered green methods, though limited studies report utilizing them with pure water or other green solvents [10,11,12]. Unlike SFE, these methods reduce volatile organic compounds but may not prevent degradation phenomena [10].

Safety concerns with essential oil extracts necessitate careful dosage evaluation because of potential toxicity. In this context, cold ethanol extractions of commercial chamomile products have demonstrated cytotoxic effects on Caco-2 cells at high concentrations, suggesting that herbal medicines are not always inherently safe [13]. Conversely, aqueous extracts obtained through conventional methods like hydrodistillation demonstrated antioxidant activity and reduced risk because of lower essential oil content and the predominant amount of various caffeoylquinic derivatives [14]. Several studies demonstrated and supported that extraction conditions significantly affect the bioactivity and toxicity of chamomile extracts.

Sotiropoulou et al. evaluated the antioxidant activity (DPPH assay), the toxicity, and the total polyphenolic content (Folin–Ciocalteu assay) of different phenolic profiles from aqueous extracts of chamomile and sage; they did not detect any total polyphenolic content in chamomile extracts at 25 °C. The toxicity tests towards *Vibrio fischeri* revealed that chamomile extracts at 80 °C (the only extraction condition characterized by LC-DAD-MS) and 100 °C have high toxicity, while the aqueous extract at 25 °C shows lower toxicity [15]. Sub-critical water extractions and the UHPLC-HESI-MS/MS characterization performed by Cvetanović and colleagues revealed varied benefits and drawbacks of extracts obtained at different temperatures, emphasizing the importance of controlling degradation to modulate bioactivity [16].

Additionally, research by Catani et al. suggested that highly glycosylated polyphenolic derivatives, monitored using HPLC-DAD, exhibit lower bioavailability compared with aglycones, despite showing similar effectiveness in counteracting Reactive Oxygen Species (ROS) generation, likely through distinct mechanisms [13].

Thus, given the lack of detailed data on the polyphenol profile of cold aqueous extracts and the absence of multivariate analyses to evaluate the effect of extraction variables under mild conditions, we designed a study to optimize cold aqueous extraction systematically. Building on existing research highlighting the safety of mild water-based extractions and the potential bioactivity of glycosylated compounds, our goal was to develop a safe and environmentally friendly method that preserves the native composition of chamomile’s water-soluble compounds using chemometric techniques.

A full factorial design was employed to evaluate the effect of extraction variables on the polyphenol profile using HPLC-DAD analysis. HPLC analysis was conducted to gather information on the polyphenol profile of chamomile at working temperatures between 15 °C and 25 °C, a range for which data are not readily available in the existing literature. Simultaneously, UV-Vis analysis was performed to assess the impact of experimental factors on spectroscopic variables through Analysis of Variance (ANOVA)-Simultaneous Component Analysis (ASCA). UV-Vis spectra, which can be quickly and cost-effectively obtained, have indeed proven valuable for characterizing bioactive compounds when combined with chemometric techniques [17,18,19]. Eventually, the antioxidant activity of the extracts was assessed using the DPPH assay coupled with a central composite design to determine if the experimental variables that maximize the polyphenol profile could also enhance the antioxidant property. Therefore, based on the literature background, this study provides an overview of how various chemometric approaches can be employed to improve extract quality and characterize the influence of process variables in a conventional cold water-based extraction process of chamomile.

## 2. Results

### 2.1. HPLC Analysis

**Figure 1:**
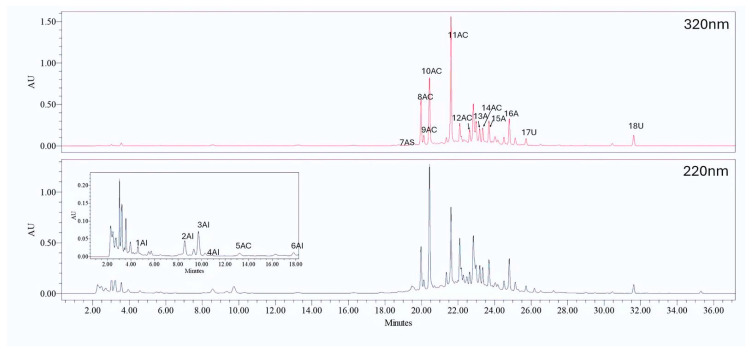
Chromatogram obtained from the HPLC analysis of the chamomile extract. The figure shows the chromatogram extracted at two different wavelengths, 220 nm (**bottom panel**) and 320 nm (**top panel**). The inset in the bottom panel provides a detailed view of the peaks in the early elution times. The peaks are labeled with abbreviations corresponding to the DOE response vectors, where AI stands for hydroxybenzoic acids, AC for hydroxycinnamic acids, and A for apigenin derivatives.

Figure 1 shows an example of a chromatogram from the HPLC analysis of a chamomile extract. In the figure, the monitored 18 chromatographic peaks are flagged with abbreviations indicating the elution order and the polyphenolic class to which they belong based on the UV-Vis spectra associated with each peak and the available analytical standards.

Specifically, only well-resolved chromatographic areas were collected at their maximum absorbance and considered as response variables to enhance. Table 1 provides a tentative attribution of the 18 chromatographic response variables based on standards and literature references. Given that the UV-Vis profiles can be easily recognized and attributed to specific polyphenolic families, we employed available aglycone standards to accurately assign the chromatographic signals to their corresponding polyphenol classes. After determining the class, we attempted to identify the specific derivative compound by consulting the literature. This involved considering only the order of elution and the compounds’ absorption maxima since the samples were not subjected to any mass spectrometry analysis.

**Figure 2:**
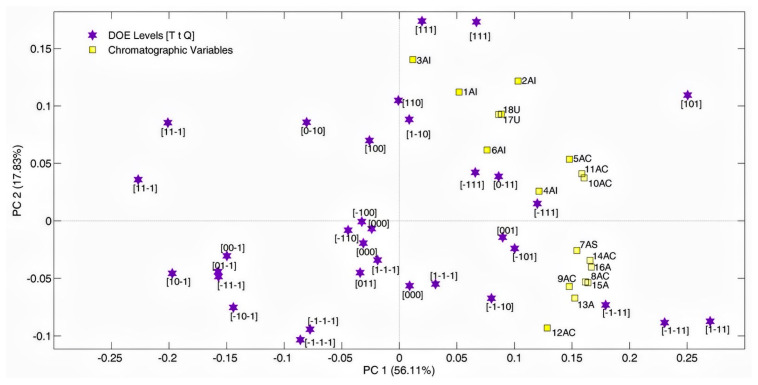
PCA for the DOE matrix and the corresponding biplot. Samples are depicted as purple stars with their respective levels [T t Q], while chromatographic variables are shown as yellow squares.

To find a simple and practical approach to optimize the amount of the considered compounds, Principal Component Analysis (PCA) was performed on the autoscaled data matrix consisting of 33 rows, which represent the produced chamomile extracts (i.e., the DOE samples), and 18 chromatographic variables. Figure 2 reports a biplot obtained by projecting the scores and loadings onto the same subspace. Inspecting the biplot allowed us to evaluate correlation trends among the samples and identify a single DOE response. Firstly, it was observed that most of the replicate samples sufficiently cluster since they tend to be closer in the PC1-PC2 subspace than the samples obtained by varying levels of the experimental variables.

All 18 chromatographic areas displayed positive loadings on PC1 (yellow squares), indicating that samples with high positive scores on PC1 contain higher amounts of all assessed compounds than the samples with lower or negative scores on this axis, which accounts for 56.11% of the total variance. Consistent with this, most samples prepared with the maximum amount of chamomile show positive scores, while those with the minimum amount exhibit negative values on PC1. PC2, which explains roughly one-third of the variance explained by PC1, reflects differences in the composition of the extracts and distinguishes samples based on their relative amounts of polyphenolic compounds. According to the biplot analysis, the projection of the samples on PC1 (scores on PC1) was selected as the single response variable for optimization. Given that nearly all the response variables, except 1AI and 3AI, display high positive scores on PC1, the linear combination of chromatographic areas represented by PC1 can be considered an indicator of the overall quality of the extract. In this particular case, the best extract is easily identified in sample C11 [1-11], which has the highest positive score on PC1. This corresponds to the vertex of the experimental domain with the highest temperature (25°), quantity (2.5 g), and minimum time (32 min). Despite this, a regression model was constructed to further explore and understand the effects of the experimental variables within the studied range.

**Figure 3:**
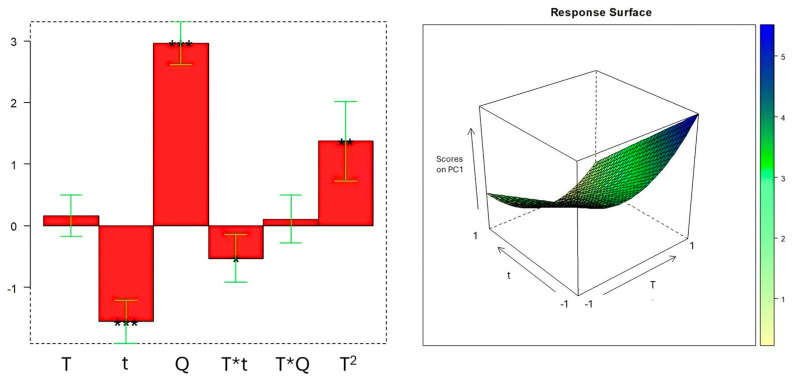
Coefficient bar plot and response surface related to the DOE empirical model. The bar plot shows the magnitude and the correlation (positive or negative) of the coefficients with respect to the response (scores on PC1). The brackets represent the confidence intervals at *p* = 0.05, and the stars indicate the significance of the coefficients (* = *p* < 0.05, ** = *p* < 0.01, *** = *p* < 0.001). The response surface graphically depicts the predicted response trend as time and temperature change, keeping the quantity fixed at the maximum level.

Figure 3 presents a bar plot illustrating the magnitude and direction (positive or negative) of the effects, along with their statistical significance. Additionally, the response surface is shown to visualize the predicted response in the experimental domain. It is important to emphasize that all non-significant terms that could be removed following the hierarchy rules for variable elimination [22] (i.e., (t^2^), (Q^2^), and (t*Q)) were eliminated from the model to reduce overfitting.

By looking at the bar plot, we can confirm that only quantity (Q) and time (t) showed a significant linear effect on the response, whereas temperature (T) affected the response through the second-order term and its interaction with time. The response surface graphically pinpointed the optimum extraction conditions at T = 25 °C, t = 32 min, and Q = 2.5 g, which corresponded to a score higher than 5. It may be necessary to specify that this value does not correspond to the one shown in Figure 2 for the PC1 axis because the biplot is the result of scaling that allows scores and loadings to be displayed simultaneously on the same subspace without changing sample distances. Table 2 provides the results of the ANOVA for the regression and the lack of fit, offering a comprehensive assessment of the model’s performance and reliability.

The model yields an adjusted R^2^ of 82.87%. According to the ANOVA table (Table 2), the very low *p*-value (<0.0001) indicates that the regression model is highly significant, explaining a substantial portion of the variability in the data. This suggests that the mathematical model fits the experimental data well. However, the model exhibited a marginally significant lack of fit (*p*-value = 0.045). Using this model, we assessed the influence of experimental factors on the polyphenolic profile of chamomile extracts, which would require mass spectrometry for proper identification. Given this, we chose to continue this study by further characterizing these extracts using a cost-effective and rapid untargeted UV-Vis analysis method.

### 2.2. UV-Vis Analysis

**Figure 4:**
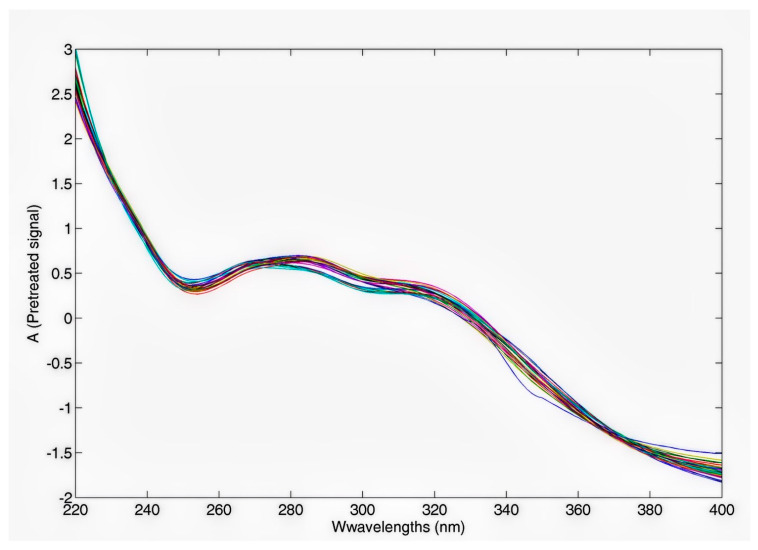
The SNV pre-treated spectra for all the produced extracts.

The UV-Vis analysis was performed on the same extracts obtained based on a complete and balanced full factorial design. Figure 4 shows the UV-Vis spectra of the chamomile extracts, each diluted 140-fold, over the wavelength range of 220 nm to 400 nm. The spectra were pre-treated according to Standard Normal Variate (SNV) to reduce scattering and baseline shift effects, ensuring a more accurate and reliable analysis.

More specific comments on the absorptions and interpretation of the multivariate signal will be given following the application of ANOVA–Simultaneous Component Analysis (ASCA), which helped understand the impact of experimental factors on the spectroscopic profile.

**Figure 5:**
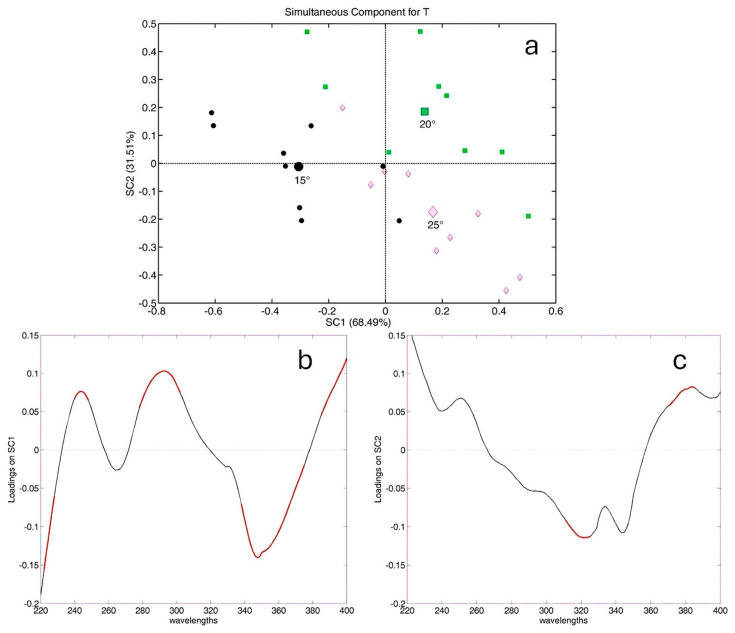
ASCA outcome for temperature: (**a**) score plot for the temperature factor; all symbols are differentiated according to the levels (15 °C, 20 °C, and 25 °C) of the extraction temperature. Spectroscopic variables significantly influenced by temperature are highlighted in red on the loading profile for the loading plot on SC1 (**b**) and SC2 (**c**).

Figure 5 reports the ASCA outcomes for the effect of temperature; in particular, panel (a) shows the score plot related to the experimental factor of temperature, which accounts for 16% of the total variance in the data. In the figure, each larger symbol represents the average effect for each temperature level, while the smaller symbols indicate the residual of each sample compared to the average effect.

The score plot reveals a distinct cluster for the extracts at 15 °C, which fall at negative scores on SC1, compared with the extracts at 20 °C and 25 °C, which fall at positive scores. This indicates a significant difference in the spectroscopic profiles based on the extraction temperature. By examining the loadings on SC1 (panel (b)), we can identify the spectroscopic variables significantly influenced by temperature (highlighted in red), which were evaluated through 1000 cycles of bootstrapping. These variables exhibit higher intensity for the extractions at 15 °C when they have negative loadings, i.e., those in the range between 330 and 370 nm. This outcome suggests that lower temperatures are more effective at solubilizing flavones and flavonols, which are likely highly glycosylated to show good solubility in water.

On the contrary, most of the extracts at 20 °C and 25 °C fall at positive scores on SC1, suggesting a higher intensity in the absorbance around 250 nm and 290 nm (positive loadings on SC1), which are wavelengths characteristic of hydroxybenzoic acid derivatives. Additionally, according to panel (c), extracts at 25 °C, which fall at negative scores on SC2, appear to be richer than those at 20 °C in hydroxycinnamic acid derivatives, as evidenced by the significant negative loadings on SC2 centered at 320 nm. Thus, it can be concluded that the temperature of extraction considerably impacts the polyphenolic profile of chamomile extracts. The extraction at 15 °C favored the extraction of glycosylated flavones and flavonols, while higher temperatures (25 °C) enhanced the extraction of hydroxybenzoic and hydroxycinnamic acid derivatives.

**Figure 6:**
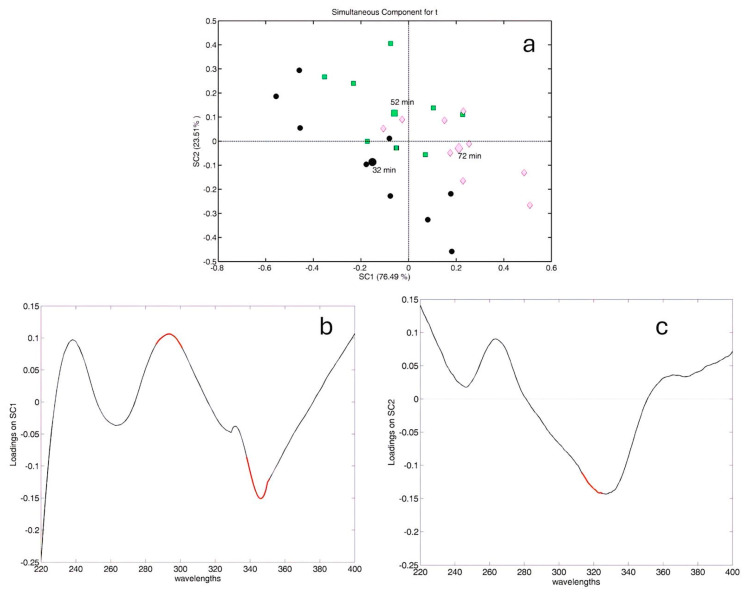
ASCA outcome for the time factor: (**a**) score plot for the time factor; all symbols are differentiated according to the different levels (32 min, 52 min, 72 min) of extraction time. Spectroscopic variables significantly influenced by time are highlighted in red on the loading profile for the loading plot on SC1 (**b**) and SC2 (**c**).

Regarding the effect of extraction time, as shown in Figure 6, we can state that extracting chamomile for 32 and 52 min yields extracts rich in flavonoids, as indicated by the negative loadings along SC1 (highlighted in red in panel (b)), compared with the samples extracted for 72 min, which instead contain a higher amount of hydroxycinnamic acids (positive loadings). However, SC2 (panel (a)) differentiates the samples extracted for 32 min from those extracted for 52 min, with the first being richer in hydroxycinnamic acids (highlighted in red in panel (c)) than the samples extracted for 52 min. Thus, according to ASCA and UV-Vis analysis, the optimal conditions reported in the previous section were interpreted as follows: 25 °C is better than 20 °C since it is richer in hydroxycinnamic acid derivatives, and this assumption could help interpret the negative second-order term for temperature (see Figure 3). However, 15 °C and 25 °C generate extracts with different relative amounts of polyphenolic compounds (15 °C in flavones and flavonols and 25 °C in hydroxybenzoic derivatives). Thus, the maximum amount of chamomile extracted at room temperature (25 °C) and for 32 min appears to be the best compromise for a high-quality extract characterized by a good amount of the main polyphenolic classes found in chamomile such as flavonols (higher in 32 min extracts), hydroxycinnamic acids, and hydroxybenzoic acids.

### 2.3. DPPH Assay

The DPPH assay was conducted to evaluate the antioxidant activity of chamomile extracts prepared under optimized conditions. This analysis also examined the impact of various experimental factors and their interactions on antioxidant properties, specifically for samples extracted using cold water. The recorded percentages of absorbance decrease for different concentrations of the optimal extract (pinpointed in Section 2.1) are as follows:

- For 1.50 mg⋅mL^−1^: 25% ± 3%.
- For 1.88 mg⋅mL^−1^: 28% ± 1%.
- For 3.00 mg⋅mL^−1^: 36.6% ± 0.3%.
- For 3.75 mg⋅mL^−1^: 50% ± 3%.
- For 7.50 mg⋅mL^−1^: 90.5% ± 0.3%.

From these data, a least squares regression was performed with an R^2^ value of 0.992. The IC50 value was obtained through interpolation, resulting in 3.85 mg⋅mL^−1^. This indicates that a chamomile solution at a concentration of 3.8 ± 0.3 mg⋅mL^−1^ (estimation and standard error of the estimate) can inhibit 50% of the oxidative capacity of DPPH.

Eventually, a central composite design (CCD) was employed to assess the effectiveness of antioxidant activity associated with the experimental variables (see Table 3).

**Figure 7:**
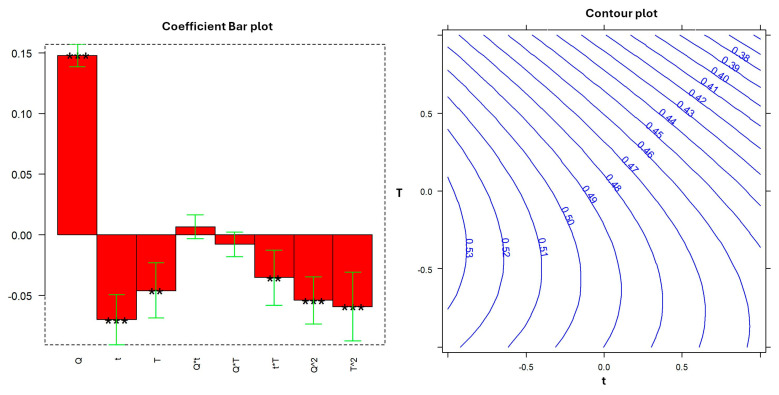
Coefficient bar plot and contour plot related to the DOE empirical model. The bar plot shows the magnitude and the correlation (positive or negative) of the coefficients with respect to the response ($t_{0}-t_{1}$). The brackets represent the confidence intervals at *p* = 0.05, and the stars indicate the significance of the coefficients (** = *p* < 0.01, *** = *p* < 0.001). The trend of the predicted response in the experimental domain is reported in the contour plot related to the maximum level of quantity (2.5 g).

The outcome produced an adjusted R^2^ of 98.59%, indicating a good fit. Figure 7 reports the effects of the experimental variables on the response (coefficient bar plot) and the predicted response within the experimental domain (contour plot). In this case, the significance of the coefficients was evaluated according to the pooled variance and six degrees of freedom. The linear terms appeared highly significant, except for temperature (T), which had a *p*-value of less than 0.01. The second-order term for time was not significant, whereas the second-order terms for temperature and quantity were highly significant and negative. This suggests a saturation effect for the quantity and indicates that higher temperatures (35 °C) did not improve the antioxidant activity of the extract. Additionally, we observed a negative effect of the interaction factor (Tt), indicating a correlation between these two experimental variables. The optimal temperature is not independent of the extraction time; thus, within the range studied, higher temperatures should be paired with shorter extraction times to achieve comparable antioxidant activity.

The model was validated by checking its prediction ability. We performed the DPPH assay with the extract obtained at 25 °C for 32 min using 2.5 g in 100 mL (an extraction condition not considered by the DOE and for model calibration). The response was 0.51 ± 0.03, where the confidence interval of the predicted response at 95% probability was between 0.051 and 0.055. Figure 7 shows the contour plot obtained by fixing the quantity at the maximum level. The interaction between temperature and time (Tt) can be seen here, showing that it is possible to achieve the same antioxidant activity at different temperatures by varying the time. The decision to modify the experimental domain was supported by the results depicted in Figure 7, which demonstrate that the same antioxidant activity, including the highest observed one, can actually be achieved across a wide temperature range by appropriately adjusting the extraction time. Ultimately, the maximum antioxidant activity was found to occur with the maximum amount of chamomile, an extraction time of approximately 32 min, and a temperature range between 18 °C and 26 °C.

## 3. Discussion

Despite substantial research on chamomile and numerous studies on optimization for industrial applications, there remains a significant gap in the use of experimental design (DOE) for this purpose [10,23]. This study addresses this gap by applying a multivariate approach to optimize cold water extraction conditions, aiming to preserve native water-soluble compounds in chamomile. Few studies have explored this topic using a multivariate approach. For example, Polcaro et al. [12] evaluated the similarity among different conventional and non-conventional water- and solvent-based extractions through PCA, while Oktaviani and co-workers [21] used DOE to optimize tryptophan extraction from chamomile in a methanol/water solvent by varying temperature, composition, and ultrasonic power under fixed time; these conditions are not directly comparable with the present work. The results obtained by the HPLC preliminary analysis confirm that it is possible to obtain a polyphenolic profile under mild conditions, contrary to what is reported in several studies (e.g., Sotiropoulou et al. [15]). Additionally, it was found that a good extract could be obtained at room temperature with four times less extraction time and concentration than what was reported by Ayhan [23], suggesting that temperature, time, and their interaction significantly impact the polyphenolic profile of chamomile extracts. The ASCA applied to UV-Vis spectroscopic profiles provided further qualitative insights. This approach is the first to use multifactorial and multivariate untargeted data to characterize and assess the effects of experimental variables on the spectroscopic profile of aqueous chamomile extracts. As a result, no existing method in the literature directly compares to our findings. However, ASCA-based insights can be compared to the results reported by Zlabur et al. [10]. They investigated the effects of solvent, time, and temperature on both conventional and ultrasound-assisted extraction. They examined a specific range of temperatures (21.4 °C, 40 °C, and 60 °C) and extraction times (5, 15, 25, and 35 min) using water as the solvent, monitoring several response variables. However, likely because of the different focus of their study, they did not develop any empirical models to assess the influence of time and temperature on these responses statistically or graphically. Based on their findings, extracts processed at 21.4 °C for 35 min showed comparable antioxidant activity to those extracted at 40 °C for 15 or 25 min and higher activity than extracts at 60 °C for 35 min. Moreover, the total non-flavonoid compounds appeared to be higher in extracts at 21.4 °C for 25 and 35 min compared with those at 60 °C for 35 min. Thus, these outcomes seem consistent with our results in highlighting the intercorrelation between time and temperature on the final extract composition. However, contrary to our findings, Zlabur et al. [10] observed that the total flavonoid content increased with temperature and time, an outcome not observed within the range of conditions we studied. This inconsistency may reflect the lower sensitivity of the method used to quantify the total flavonoid fraction, described in reference [24], to glycosylated derivatives, which tend to decrease in favor of their respective aglycones as the temperature increases.

The DPPH assay results confirmed that chamomile extracts prepared at 25 °C for 32 min with 2.5 g of dry chamomile showed substantial antioxidant activity, achieving an IC50 value of 3.8 mg⋅L^−1^. Since IC50 values are highly dependent on the specific experimental protocols used, direct comparisons with other studies may not be entirely consistent. Additionally, most reported IC50 values are based on the weight of the dry extract, whereas, in this study, we chose not to subject the extract to further processing or heat treatment, instead expressing the IC50 in terms of the concentration of dry chamomile. Within this context, the observed IC50 values are comparable to the EC50 values reported by Baranauskienė for dry water residues from hydrodistillation (0.59–3.08 mg⋅L^−1^), which, as in our study, were found to be rich in dicaffeoylquinic acid derivatives [25].

Moreover, given the lack of a clear correlation between total phenolic compounds and antioxidant activity, as well as the conflicting results reported in the literature [16,17], we opted for a central composite design with expanded ranges relative to the full factorial design. This approach was used to evaluate the correlation of experimental variables with antioxidant properties and, indirectly, with the polyphenolic profile. The results indicate that extracts with varying relative amounts of bioactive compounds, as highlighted in Section 2.1 and Section 2.2, likely exert similar levels of antioxidant activity.

## 4. Materials and Methods

### 4.1. Sample Treatment

Using the Tube Mill 100 control (IKA, Staufen, Germany), commercially available dried chamomile flower heads from the same lot were ground for 90 s at 5000 rpm. After grinding, the chamomile was sieved with a 1 mm mesh sieve and weighed in such a quantity as to obtain, after adding 100 mL of Milli-Q water (Millipore, Bedford, MA, USA), different mass-to-volume ratios. Each extract was maintained at a constant temperature (T) using a thermostated circulating water bath and subjected to magnetic stirring for a well-defined extraction time (t). The extract was centrifuged for 10 min at 3000 rpm and then filtered using CA filters 0.22 µm, 13 mm. The resulting solution was analyzed through the HPLC apparatus. Eventually, UV-Vis analysis was performed only after 140-fold dilution of the samples.

### 4.2. HPLC Analysis

The qualitative analysis of the aqueous extracts was performed using HPLC (Waters, Milford, MA, USA) equipped with a Kinetex C18 100 Å column (Phenomenex, Torrance, CA, USA), a Model 600 pump, a pump controller module 600, a 717 Plus autosampler, and a series photodiode array detector 996. The chromatographic system was controlled by Empower Pro software (Waters, Milford, MA, USA, https://www.waters.com/waters/en_US/Empower-Software-Solutions/nav.htm?cid=513188&locale=en_US, accessed on 11 October 2024). Chromatographic analysis was carried out at a constant flow rate (1 mL⋅min^−1^) under gradient elution conditions with an injection volume of 10 µL. The mobile phase used was degassed using an Agilent 1200 degasser (Agilent Technologies, Waldbronn, Germany) and eluted in gradient mode consisting of the following solutions: Solution A, 0.1% H_3_PO_4_ (HPLC-grade, Thermo Scientific Chemicals (Waltham, MA, USA)) in Milli-Q water and Solution B, 100% acetonitrile (≥99.9%, gradient-grade, Sigma-Aldrich). Then, 10 min of isocratic elution (5% B) was followed by 30 min of gradient from 5% to 95% of B and to an isocratic 95% B for the other 10 min and recondition at 5% B, for a total run time of 60 min. For qualitative analysis, HPLC-grade standards obtained from Sigma-Aldrich (St. Louis, MO, USA) were analyzed under the same HPLC conditions used for the chamomile extracts. The standards included the following:

- Hydroxybenzoic acid derivatives: Protocatechuic acid (>97.0% purity), Gentisic acid (98.0% purity), Syringic acid (>95.0% purity), Gallic acid (97.5–102.5% purity), and Vanillic acid (>97.0% purity).
- Hydroxycinnamic acids: Caffeic acid (>98.0% purity), trans-Ferulic acid (99.0% purity), *p*-Coumaric acid (>98.0% purity), and 3-O-Caffeoylquinic acid (95.0% purity), Syringic acid (purity greater than 95.0%), Gallic acid (97.5–102.5% purity).
- Flavones: Apigenin (95.0% purity) and Luteolin (>97.0% purity).
- Flavonols: Quercetin (>95.0% purity) and Rutin trihydrate (>95.0% purity).

Additionally, Tyrosol (98.0% purity) was included as a standard. Stock solutions were prepared at a concentration of 1 mg⋅mL^−1^ by dissolving the standards in a mixture of acetonitrile and methanol (≥99.0%, HPLC grade, Carlo Erba Reagenti, Milan, Italy) in a 70:30 ratio. From these stock solutions, diluted standard solutions at a concentration of 5 mg⋅L^−1^ were prepared and analyzed. Though certain standards were not found in the chamomile extracts, their inclusion significantly aided in understanding retention behavior, facilitating the interpretation of chromatographic profiles. While flavonols were detected in the chamomile extracts, they were not fully resolved and thus were not considered in the final analysis.

### 4.3. UV-Vis Analysis

The aqueous chamomile extracts, prepared according to the procedure described in Section 4.1, were analyzed using UV-Vis spectroscopy with a single beam spectrophotometer, ONDA UV-30 SCAN UV (Onda, Carpi, MO, Italy). The spectrophotometer was equipped with a deuterium lamp for the UV region, a tungsten halogen lamp for the visible region, and a silicon photodiode detector. Quartz cuvettes with a 1 cm optical path length and Milli-Q water as the reference blank were used. The spectra were acquired within a spectral range of 200–500 nm with a resolution of 1 nm. The same instrument was used for the DPPH colorimetric assay, in which a single wavelength was monitored by running, in this case, the blank in a methanol solution (methanol ≥ 99.9%, HPLC grade, Sigma-Aldrich).

### 4.4. DPPH Assay

The DPPH assay was selected because of the stability of the 2,2-diphenyl-1-picrylhydrazyl free radical, which is commonly used as a reagent for analyzing antioxidant activity. To evaluate the antioxidant properties, each extract was diluted two-fold and reacted with the DPPH solution. Thus, a working solution of 0.025 mg⋅mL^−1^ was prepared from the stock solution of DPPH 0.5 mg⋅mL^−1^ in methanol. To proceed with the assay, 4.35 mL of the working DPPH solution was withdrawn and diluted to a volume of 5 mL with methanol and analyzed to obtain the $t_{0}$ absorbance (55 µM; $t_{0}\;$= 0.532 ± 0.015). The reacted solution was obtained by adding 150 µL of the diluted extract to 4.35 mL of the DPPH working solution in a 5 mL volumetric flask. Both solutions were left to react in the dark for one hour, after which the UV-Vis spectra were recorded for both $t_{1h}$ and ${t_{0}}^{\prime }$ at 516 nm (to check the stability of the DPPH reagent after one hour without the reacting sample). The difference in absorbance at 516 nm between the mean $t_{0}$ and $t_{1h}$ was considered as the response to be optimized according to DOE. The IC50 value for the optimal chamomile extract was determined using the established procedure. To calculate the IC50, five different dilutions of the extract (two replicates each at concentrations of 1.5 mg⋅mL^−1^, 1.88 mg⋅mL^−1^, 3.0 mg⋅mL^−1^, 3.75 mg⋅mL^−1^, and 7.5 mg⋅mL^−1^) were prepared and subjected to the DPPH assay. The absorbance values obtained from the two replicates were averaged, and this mean value was used in the final IC50 calculation. The percentage decrease in absorbance (A_DPPH%_) was calculated using the following equation:

(1) $$A_{\mathrm{DPPH}\%}=\frac{\left(t_{0}-t_{1}\right)}{t_{0}}\times 100$$

where $t_{0}$ is the absorbance value of the DPPH working solution at time zero (without the addition of the chamomile solution) and $t_{1}$ is the average absorbance of the two replicates recorded after the samples reacted with DPPH for one hour. We chose not to apply additional processing or heat stress to the extract. Therefore, the IC50 is expressed in terms of the concentration of dry chamomile, reflecting the extract’s antioxidant potential based solely on its dry weight without any alteration from external processing factors.

### 4.5. DOE-Based Sample Preparation

**Figure 8:**
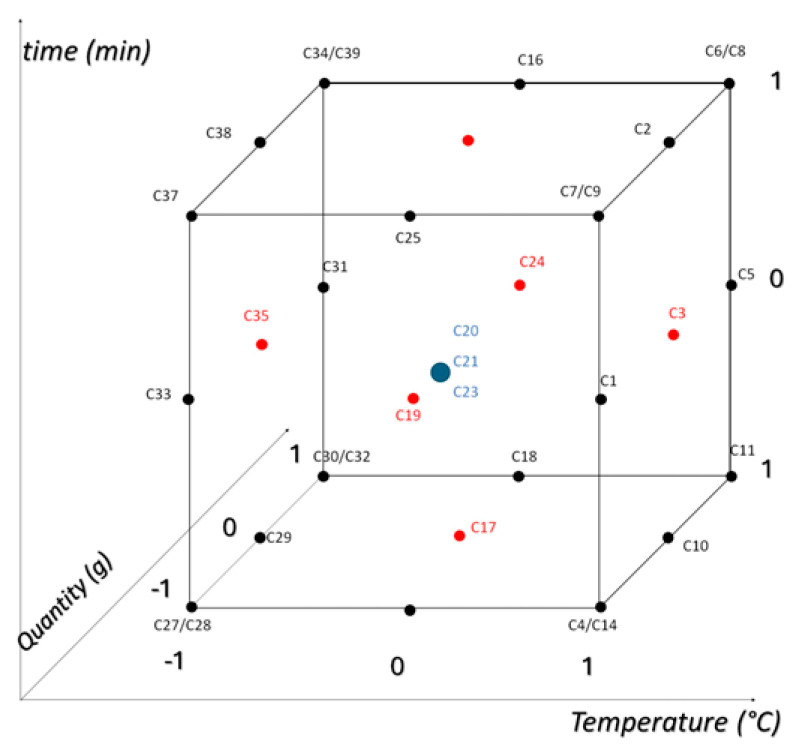
Explored experimental domain, sample names, and relative scaled levels. The different colors represent the central point (in blue) and the points at the center of each face of the cube (red). This color differentiation helps visualize the experimental design and understand the spatial arrangement of the samples within the experimental domain.

For the HPLC preliminary analysis, the temperature (T) was varied from 15 °C to 25 °C, the time (t) from 32 min to 72 min, and the chamomile quantity (Q) from 1.5 g to 2.5 g, as shown in Figure 8.

**Figure 9:**
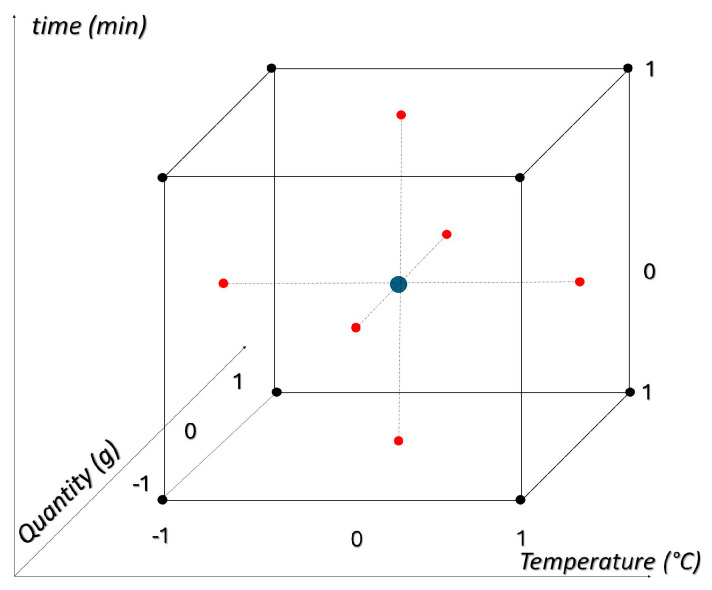
Explored experimental domain through a face-centered design and relative scaled levels. The different colors represent the central point (in blue) and the points at the center of each face of the cube (red).

Figure 9 illustrates the experimental domain and the samples obtained by simultaneously and randomly varying the experimental factors, along with their respective replicates. It can be seen that two samples expected by the model are not reported in the experimental domain ([0-1-1] and [0 1 0]). This is because the chromatographic runs were wrong and aborted. Given the spatial arrangement of these points, it was not necessary to repeat the measurements (a decision made to save time and solvents), as the orthogonality of the design was not drastically affected (the variance inflation factor, which is a measure of multicollinearity among the independent variables in a multiple regression model, was slightly above 1). A total of 33 extracts were analyzed using an HPLC-DAD apparatus.

A central composite design, with expanded ranges compared with the full factorial design, was selected to evaluate the influence of experimental factors on the antioxidant activity of aqueous cold extracts. In this case, the decision was made to expand the experimental domain because of the limited variability observed in antioxidant activity within the previous ranges. Additionally, the number of experiments was reduced compared with the previous design.

In detail, the range for quantity was widened to investigate lower weight/volume ratios. For the time factor, longer durations (up to 92 min) were explored, and for the temperature variable, higher values that could still be considered cold (up to 35 °C) were investigated. The response Y was calculated by subtracting the mean absorbance of DPPH at $t_{0}$ from the absorbance of the reacted sample at t = 1 h.

### 4.6. Statistical Analysis

#### 4.6.1. Design of Experiment

Experimental Design (DOE) was employed to explore an entire experimental domain systematically and obtain an empirical mathematical model able to predict the response in the whole experimental domain [26].

A three-level full factorial design was employed to consider all possible combinations of factors. In addition, the same factors were investigated through a face-centered design that provided full coverage of the experimental space with fewer experiments than a full factorial design. For both the selected experimental designs, replicates were performed at the vertices and center of the experimental domain, and a second-order predictive model, reported in Equation (2), was postulated as follows:

(2) $$Y=b_{0}+b_{1}X_{1}+b_{2}X_{2}+b_{3}X_{3}+b_{12}X_{1}X_{2}+b_{13}X_{1}X_{3}+b_{23}X_{2}X_{3}+b_{11}X_{1}^{2}+b_{22}X_{2}^{2}++b_{33}X_{3}^{2}$$

Non-significant linear terms were retained to maintain model consistency during origin shifts or axis rotations. Non-significant higher-order terms were always removed, whereas interaction terms were eliminated only if their corresponding second-order terms were also removed, ensuring the model remained well-defined and consistent under transformations [22]. The outcomes were interpreted in terms of significant influencing factors and through a response surface (and/or iso-response plot) [26]. The significance of the estimated coefficients was performed through a *t*-test considering specific degrees of freedom (Nreplicate-1) and coefficient standard deviation S_c,_ calculated as follows:

S_c_ = (S_pooled_^2^ × C_ii_)^1/2^ (3)

where S_pooled_^2^ is the pooled experimental variance estimated through independent measurements and C_ii_ is the variance of the coefficient (the diagonal element of the variance-covariance matrix).

The Analysis of Variance, the lack of fit, and the predictive ability of the models were considered for validation.

#### 4.6.2. Principal Component Analysis

To inspect and interpret the multivariate data obtained from the designed experiments, Principal Component Analysis (PCA) was applied. PCA is an unsupervised multivariate statistical technique for dimensionality reduction that is used to facilitate the visualization of high-dimensional datasets. This technique transforms correlated variables into a set of uncorrelated directions called principal components, which capture the maximum and decreasing variance in the data. Applying PCA to a Design of Experiments (DOE) data matrix, where rows represent the number of postulated experiments and the columns represent the variables to be optimized (multiple responses), it is possible to evaluate trends among the experiments and correlations between the variables [27].

#### 4.6.3. ANOVA–Simultaneous Component Analysis (ASCA)

ANOVA–Simultaneous Component Analysis (ASCA) was employed to conduct an Analysis of Variance (ANOVA) within a complex multivariate framework [28]. ASCA is typically used to confirm the significance of factors by decomposing the experimental matrix into individual multivariate matrices that reflect the effects of each design term, following the ANOVA methodology.

The model used in ASCA can be represented as follows:

X = Xα + Xβ + Xγ + Xαβ + Xαγ + Xβγ + XE(4)

where Xα, Xβ, and Xγ represent the matrices accounting for the main effects of the factors. Xαβ, Xαγ, and Xβγ capture the interaction effects between the factors. Eventually, XE represents the residual matrix.

The second step involves conducting Principal Component Analysis (PCA) separately on each effect matrix as follows:

***X***_*i*_ = ***T***_*i*_
***P***^*T*^_*i*_ with *i* = α,β, γ, αβ, αγ, βγ (5)

where ***T***_*i*_ and ***P***_*i*_ denote the scores and loadings matrices for each factor, respectively. The significance of these effects and loadings is assessed through permutation tests and bootstrapping procedures, respectively [29].

## 5. Conclusions

The optimal extraction conditions identified (25 °C, 32 min, and 2.5 g of chamomile) produced extracts rich in polyphenolic compounds such as hydroxybenzoic acids, hydroxycinnamic acids, and flavonol derivatives. This represents an ideal compromise for extracting the major water-soluble polyphenolic classes.

UV-Vis analysis, functioning as an untargeted technique, provided an overview of absorptions influenced by experimental variables without the need to identify individual compounds. The distinctive UV-Vis spectra for various polyphenolic classes made this method particularly effective for qualitative assessments and interpreting compositional trends in the extracts.

The presented chemometric analyses highlighted the significant impacts of temperature, time, and their interaction on the polyphenolic profile. Lower temperatures favored the solubilization of glycosylated flavones and flavonols, while higher temperatures enhanced the extraction of hydroxybenzoic and hydroxycinnamic acids. As a result, combining an extraction at 25 °C with a shorter extraction time yields extracts with greater antioxidant activity.

The antioxidant activity of the extracts was confirmed through the DPPH assay, showing an IC50 value of 3.8 mg of dry chamomile⋅L^−1^. Within the studied range, comparable antioxidant activity can be achieved by appropriately adjusting temperature and time. This suggests that extracts with slight compositional differences can still exhibit similar antioxidant properties because of synergistic effects among polyphenolic compounds when tested using the DPPH assay. Furthermore, a future perspective of the present work is represented by the possibility of applying a targeted anti-inflammatory assay, such as COX inhibition, that might yield complementary insights specific to chamomile’s anti-inflammatory activity.

A noteworthy consideration is the strong influence of environmental factors such as pedoclimatic conditions, disease and pest exposure, seasonal variations, drying, and storage. These factors significantly impact the native composition of medicinal herbs. In this study, such variability was minimized by using a single batch of dried commercial chamomile, focusing on the effects of the experimental variables.

A further limitation that needs to be noted is that contrary to hot beverages, which can reduce the risk of gastrointestinal infections by eliminating potentially harmful microorganisms present in the herbal material, the cold water extraction method does not offer the same microbial reduction benefit.

In conclusion, this study illustrates how various chemometric approaches can enhance extract quality and characterize the influence of process variables using a simple model of conventional cold water-based extraction of chamomile.

## Figures and Tables

**Table 1:** A tentative attribution based on non-glycosylated standards and literature references [7,8,14,20,21], where D1 and D2 are the first and second (elution order) derivatives.

Code	Compound
1AI	*p*-hydroxybenzoic acid ^3^
2AI	Gentisic Acid ^1^
3AI	Protocatechuic Acid ^1^
4AI	Tyrosol/Tryptophan Derivative ^2^
5AC	3-O-Caffeoylquinic acid ^1^
6AI	*p*-hydroxybenzoic acid derivative ^2^
7AS	Salicylic acid derivative ^3^
8AC	5-O-Caffeoylquinic acid ^2^
9AC	4-O-Caffeoylquinic acid ^2^
10AC	*cis*-2-Hydroxy-4-methoxycinnamic acid 2-O-glucopyranoside ^3^
11AC	*tran*-2-Hydroxy-4-methoxycinnamic acid 2-O-glucopyranoside ^3^
12AC	Dicaffeoylquinic acid D1 ^3^
13A	Apigenin-7-O-glucoside ^2^
14AC	Dicaffeoylquinic acid D2 ^3^
15A	Apigenin-7-O-hexoside D1 ^2^
16A	Apigenin-7-O-hexoside D2 ^2^
17U	Unknown
18U	Unknown

^1^ Standard-based attribution. ^2^ Tentative attribution based on aglycone standards and the literature. ^3^ Literature-based tentative attribution.

**Table 2:** ANOVA table for the regression and lack of fit. The degrees of freedom (DF), F-ratio, and *p*-value (Prob.) for F-ratio ≥ F-ratio critical are reported.

Origin	DF	Sum of Squares	Mean Squares	F-Ratio
Regression	6	253.357	42.226	26.735
Residues (E)	26	41.066	1.579	Prob
Total	32	294.423		<0.0001
Lack of Fit	18	36.421	2.023	36.421
Pooled Error	8	5.006	6.626	Prob
Residues	26	41.427		0.045

**Table 3:** DOE matrix with nominal values of the experimental factors and the response (Y), which was calculated as $t_{0}-t_{1}$. Replicates were performed at the center and vertices of the cube.

Sample	Q	T (min)	T	Y
C1	0.5	32	15	0.207
C1bis	0.5	32	15	0.228
C2	0.5	32	35	0.204
C2bis	0.5	32	35	0.224
C3	0.5	92	15	0.144
C3bis	0.5	92	15	0.126
C4	0.5	92	35	0.090
C5	0.5	62	25	0.213
C6	2.5	32	35	0.495
C6bis	2.5	32	35	0.470
C7	2.5	32	15	0.517
C8	2.5	32	15	0.517
C9	2.5	92	15	0.465
C9bis	2.5	92	15	0.456
C10	2.5	92	35	0.350
C11	2.5	62	25	0.501
C12	1.5	62	25	0.389
C12bis	1.5	62	25	0.403
C14	1.5	62	35	0.377
C15	1.5	62	15	0.407
C16	1.5	92	25	0.329
C17	1.5	32	25	0.470

## Data Availability

Data will be made available on request.
